# Triphala inhibits both *in vitro *and *in vivo *xenograft growth of pancreatic tumor cells by inducing apoptosis

**DOI:** 10.1186/1471-2407-8-294

**Published:** 2008-10-10

**Authors:** Yan Shi, Ravi P Sahu, Sanjay K Srivastava

**Affiliations:** 1 Department of Pharmaceutical Sciences and Cancer Biology Center, Texas Tech University Health Sciences Center, School of Pharmacy, Amarillo, Texas, USA

## Abstract

**Background:**

Triphala is commonly used in Ayurvedic medicine to treat variety of diseases; however its mechanism of action remains unexplored. This study elucidates the molecular mechanism of Triphala against human pancreatic cancer in the cellular and in vivo model.

**Methods:**

Growth-inhibitory effects of Triphala were evaluated in Capan-2, BxPC-3 and HPDE-6 cells by Sulphoradamine-B assay. Apoptosis was determined by cell death assay and western blotting. Triphala was administered orally to nude mice implanted with Capan-2 xenograft. Tumors were analyzed by immunohistochemistry and western blotting.

**Results:**

Exposure of Capan-2 cells to the aqueous extract of Triphala for 24 h resulted in the significant decrease in the survival of cells in a dose-dependent manner with an IC50 of about 50 μg/ml. Triphala-mediated reduced cell survival correlated with induction of apoptosis, which was associated with reactive oxygen species (ROS) generation. Triphala-induced apoptosis was linked with phosphorylation of p53 at Ser-15 and ERK at Thr-202/Tyr-204 in Capan-2 cells. Above mentioned effects were significantly blocked when the cells were pretreated with an antioxidant N-acetylcysteine (NAC), suggesting the involvement of ROS generation. Pretreatment of cells with pifithrin-α or U0126, specific inhibitors of p53 or MEK-1/2, significantly attenuated Triphala-induced apoptosis. Moreover, NAC or U0126 pretreatment significantly attenuated Triphala-induced p53 transcriptional activity. Similarly, Triphala induced apoptosis in another pancreatic cancer cell line BxPC-3 by activating ERK. On the other hand, Triphala failed to induce apoptosis or activate ERK or p53 in normal human pancreatic ductal epithelial (HPDE-6) cells. Further, oral administration of 50 mg/kg or 100 mg/kg Triphala in PBS, 5 days/week significantly suppressed the growth of Capan-2 pancreatic tumor-xenograft. Reduced tumor-growth in Triphala fed mice was due to increased apoptosis in the tumors cells, which was associated with increased activation of p53 and ERK.

**Conclusion:**

Our preclinical studies demonstrate that Triphala is effective in inhibiting the growth of human pancreatic cancer cells in both cellular and in vivo model. Our data also suggests that the growth inhibitory effects of Triphala is mediated by the activation of ERK and p53 and shows potential for the treatment and/or prevention of human pancreatic cancer.

## Background

Pancreatic cancer is a leading cause of cancer-related deaths with extremely poor prognosis [1,2]. It is estimated that about 33,000 new cases of pancreatic cancer will be diagnosed in the United States each year [3,4]. The low survival rate is due to insensitivity of pancreatic cancer to most of oncologic therapies such as chemotherapy, radiotherapy and immunotherapy [5,6]. New therapeutic strategies are therefore urgently needed to combat with this deadly form of cancer. Several epidemiological studies suggested that diet rich in fruits, vegetables or certain herbs may be protective against various human malignancies including pancreatic cancer [7-9].

Triphala (TPL) is the most commonly used Indian Ayurvedic herbal formulation, consisting equal parts of three medicinal dried plant fruits *Emblica officinalis*, *Terminalia belerica *and *Terminalia chebula*. It is an important medicine of the "Rasayana" group of Ayurveda and is believed to promote immunity, health and longevity [10]. Rich in antioxidants, Triphala, plays an essential role in the treatment of a wide variety of conditions such inflammation, anemia, constipation, asthma, jaundice, chronic ulcers and AIDS [10,11]. Gallic acid and ascorbic acid are found to be the major ingredients of Triphala [12]. Recent study suggested that Triphala significantly reduce benzo(a)pyrene-induced forestomach tumorigenesis in mice [13]. It has also been shown to suppress the growth of MCF-7 breast cancer cells and protect against radiation-induced oxidative damage [14-16]. However, the molecular mechanism of the anticancer effects of Triphala has not yet been established and its effect against pancreatic cancer not known.

In the present study, we demonstrate that Triphala significantly inhibit the proliferation of Capan-2 and BxPC-3 human pancreatic cancer cells. The apoptosis inducing effects of Triphala in Capan-2 cells was associated with the generation of reactive oxygen species, activation of ERK, P53 and caspase-3 cascade. Moreover, oral administration of Triphala significantly suppresses the growth of Capan-2 tumor xenograft which correlates with increased apoptosis and activation of p53 and ERK in the tumors, in agreement with our *in vitro *observations.

## Methods

### Chemicals and Antibodies

Triphala (TPL) was obtained from Tansukh Herbal Corporation (Lucknow, India). Anti-actin, sulforhodamine B (SRB), and N-Acetyl-Cysteine (NAC) were purchased from Sigma (St. Louis, MO). The Antibodies against phospho-H2A.X (Ser-139), caspase-9, caspase-3, cleaved caspase-3, poly (ADP-ribose) polymerase (PARP), cleaved PARP, phospho-ERK (Thr-202/Tyr-204), phospho-P53 (Ser-15), phospho-ATM (Ser-1981), phospho-MEK-1 (Ser-217/221), ERK, and P53 were purchased from Cell Signaling (Danvers, MA). The cell death detection ELISA kit was obtained from Roche Diagnostic Gmbh (Mannheim, Germany) and P53 transcription factor assay kit was procured from TransAM (Carlsbad, CA). Enhanced chemiluminescence kit was bought from Perkin Elmer Life Science Products (Boston, MA). The specific probe DCFDA was obtained from Molecular Probes (Eugene, OR). U0126 (ERK specific inhibitor), and Pifithrin-α (p53 specific inhibitor) were obtained from Calbiochem (San Diego, CA). NE-PER Nuclear and Cytoplasmic extraction reagent kit was acquired from Pierce biotechnology (Rockford. IL).

### Cell Culture

Human pancreatic cancer cell line Capan-2 and BxPC-3 were purchased from American Type Culture Collection (Rockville, MD). Capan-2 cells express wild type p53 whereas BxPC-3 cells harbor mutated p53. Monolayer cultures of Capan-2 cells were maintained in McCoy's medium and BxPC-3 cells in RPMI 1640 medium supplemented with 10% fetal bovine serum, PSN antibiotic mixture (10 ml/L) (Gibco BRL, Grand Island, NY). The cultures were maintained at 37°C in a humidified chamber of 95% air and 5% CO2. Normal human pancreatic ductal epithelial cells (HPDE-6) were a generous gift from Dr. Ming-Sound Tsao, University of Toronto, Toronto, Canada. The long term culture of pancreatic ductal epithelial cells derived from normal and benign adult human pancreata was achieved by infection with a retrovirus containing the E6 and E7 genes of the human papilioma virus 16. These cells were considered as near normal pancreatic epithelial cells. The genetic characterization and maintenance of primary culture of HPDE-6 cells were done as described previously [17,18].

### Cell Survival Assays

The effect of Triphala on the survival of Capan-2, BxPC-3, and HPDE-6 cells was determined by Sulforhodamine B assay. Briefly, 5000 cells were plated in 96 well plates and allowed to attach overnight. The medium was replaced with fresh medium containing varying concentrations of Triphala, which was dissolved in PBS and filtered through 0.22 μm before use. Plates were developed as described by us previously [19,20] and read at 570 nm using Bio Kinetics plate reader.

### Determination of Apoptosis

Apoptosis induction in control and Triphala treated cells was determined by cell death detection ELISA kit according to manufacturer's instructions. Briefly, cytoplasmic histone associated DNA fragments from control or Triphala treated cells were extracted and incubated in the microtiter plate coated with anti-histone antibody. Subsequently, after color development the absorbance of the samples was read at 405 nm using Biokinetics EL340 microplate reader.

### Generation of reactive oxygen species (ROS)

The generation of ROS was evaluated by measuring the levels of hydrogen peroxide produced in the cells by flow cytometry. Levels of hydrogen peroxide in control and Triphala treated cells was determined by staining the cells with 6-carboxy-2',7'-dichlorodihydrofluorescein diacetate (DCFDA). DCFDA is cell permeable and is cleaved by non-specific esterases and oxidized by peroxides produced in the cells to form fluorescent 2',7'-dichlorofluorescin (DCF). The intensity of DCF fluorescence is proportional to the amount of peroxide produced in the cells. Briefly, 0.5 × 10^6^ cells were plated in 25 cm2 flasks and allowed to attach overnight. After treatment of Capan-2 cells with Triphala, cells were further incubated with 5 μM DCFDA at 37°C for 15 min. Subsequently, cells were washed and resuspended in PBS and analyzed for DCF fluorescence by using a Coulter XL flow cytometer. Approximately 20,000 cells were evaluated for each sample. In all determinations, cell debris and clumps were excluded from the analysis.

### Western blot analysis

Capan-2 cells were exposed to varying concentrations of Triphala for the indicated time periods, washed twice with ice-cold PBS and lysed on ice as described by us previously [19,20]. Tumors obtained from control and Triphala treated mice were washed with cold PBS, minced and homogenized in above-mentioned lysis buffer. The cell/tumor lysate was cleared by centrifugation at 14,000 × g for 30 min. Lysate containing 60 μg protein was resolved by 10% sodium dodecyl sulfate (SDS)-polyacrylamide gel electrophoresis (PAGE) and the proteins were transferred onto polyvinylidene fluoride (PVDF) membrane. After blocking with 5% non-fat dry milk in Tris buffered saline, membrane was incubated with the desired primary antibody overnight. Subsequently, the membrane was incubated with appropriate secondary antibody, and the immunoreactive bands were visualized using enhanced chemiluminescence kit according to the manufacturer's instructions. Each membrane was stripped and re-probed with antibody against actin (1:40000 dilution) to ensure equal protein loading.

### ERK kinase activity

Control and Triphala-treated cells were lysed on ice by lysis buffer as described above. Approximately 500 μg protein lysate was incubated overnight with 15 μl immobilized antibody bead slurry at 4°C and centrifuged at 14,000 × g for 30 seconds at 4°C. Pellet was washed with PBS and resuspended in 50 μl of kinase buffer supplemented with 200 μM ATP and substrate and incubated for 30 minutes at 30°C. The protein was resolved by gel electrophoresis.

### Nuclear P53 transcription activity assay

Nuclear extract from control and Triphala treated cells was prepared using NE-PER Nuclear and Cytoplasmic extraction reagent kit. P53 transcription activity was measured by the TransAM P53 kit according to the manufacturer's instructions. Briefly, about 10 μg nuclear extract was incubated with binding buffer for 1 hour at room temperature followed by addition of p53 antibody. Subsequently, after color development the absorbance of the samples was read at 450 nm using Biokinetics EL340 microplate reader with a reference wavelength of 655 nm.

### In vivo xenograft experiment

Female athymic nude mice (NCR nu/nu) were purchased from Tacomics. The use of athymic nude mice and their treatment was approved by the Institutional Animal Care and Use Committee (IACUC), University of Pittsburgh and Texas Tech University Health Sciences Center, and all the experiments were carried out in strict compliance with their regulations. Mice were kept on antioxidant free AIN-76A special diet a week before starting the experiment. Tumor xenograft in athymic nude mice was performed as described by us previously [21]. Briefly, 1 × 10^6 ^Capan-2 cells in 0.1 ml PBS were injected subcutaneously in both the flanks of nude mice. Mice were divided randomly into three groups with 5 mice in each group. Since each mouse had two tumors, every group consisted of 10 tumors. Group 1 served as controls and received 0.1 ml PBS by oral gavage. Group 2 received 50 mg Triphala/Kg body weight five times a week (Monday-Friday), Group 3 received 100 mg Triphala/Kg five times a week (Monday-Friday) respectively in 0.1 ml PBS by oral gavage. Treatment started the same day after tumor cell implantation. Triphala was dissolved in PBS and filtered through 0.22 μm before administering to the mice. Control mice received PBS only. Tumors were measured by Vernier calipers three times a week (Monday, Wednesday, Friday) and each mouse was weighed twice a week (Monday and Friday).

### Apoptosis measurement in human tumor xenografts

Paraffin-embedded tissue sections (4 μm in thickness) were stained by hematoxylin and eosin (H&E). Apoptosis was measured by TUNEL staining kit according to the manufacturer's instructions. Briefly, tissue sections were incubated with proteinase K (20 μg/ml in 10 mM Tris-HCl, pH 7.4) for 15 min at 37°C. DNA breaks were then labeled with terminal deoxytransferase (TdT) and biotinylated deoxy UTP. Staining without TdT enzyme or the biotinylated substrate was used as negative controls. Endogenous peroxidase activity was quenched by incubating the slides in 3% hydrogen peroxide, followed by washing in PBS.

### Immunohistochemistry

Immunohistochemical staining was performed on 4 μm paraffin-embedded tissue sections using ABC avidin/biotin method. Briefly, paraffin sections were deparaffinized and rehydrated. Endogenous peroxide activity was quenched by incubating sections in xylene/ethanol for 15 min. To unmask antigens, slides were digested for 10 minutes at 37°C by using pepsin. Slides were incubated with antibodies against phospho-ERK (1:100), phospho-p53 (1:200) overnight at 4°C. After incubating with secondary antibody (1:100), immunoreactive products were developed using 3,3'-diaminobenzidine (DAB) as the chromogen with standardized development times.

### Densitometric scanning and statistical analysis

The intensity of immunoreactive bands was determined using a densitometer (Molecular Dynamics, Sunnyvale, CA) equipped with Image QuaNT software. Results are expressed as mean values with 95% confidence intervals. All statistical calculations were performed using InStat software and GraphPad Prizm 4.0. Non parametric analysis of variance (ANOVA) followed by Bonferroni post hoc multiple comparison tests were used to test the statistical significance between multiple control and treated groups. Student t test was used to compare control and treated group only. Differences were considered significant at P < 0.05.

## Results

### Effects of Triphala on the survival of human pancreatic cancer cells and induction of apoptosis

**Figure 1:**
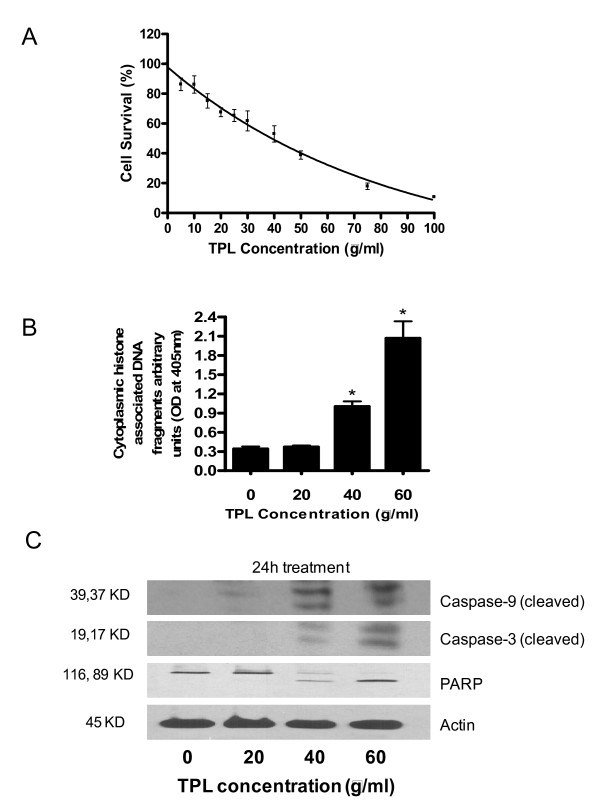
**Antiproliferative and apoptosis-inducing effects of Triphala in human pancreatic cancer cell line Capan-2.** A) Cells were cultured in the presence of varying concentrations of Triphala (5–100 μg/ml) for 24 hours. Cell proliferation was measured by Sulphorhodamine B assay with eight replicates per Triphala concentration. Results of data are derived from three independent experiments and expressed as percent survival of Triphala treated cells compared to that of PBS treated control cells. B) Cells were treated with 20, 40 and 60 μg/ml Triphala for 24 hours. Cytoplasmic histone-associated DNA fragments were determined using cell death detection kit. The data represents mean ± SD of two independent experiments with 3 replicate in each experiment. C) Cells were treated with 20–60 μg/ml Triphala 24 hours. Subsequently cell lysates were prepared and 60 μg total protein was subjected to sodium dodecyl-polyacrylamide gel electrophresis followed by immunoblot analyses. The expression of caspase-9, caspase-3 and PARP were detected using appropriate antibodies. Blot was stripped and reprobed with anti-actin antibody to ensure equal protein loading. These experiments were performed 2–3 times independently, with similar results obtained in each experiment. *Statistically different compared with PBS-treated control (P < 0.05).

We first examined the effects of Triphala on the growth of Capan-2 human pancreatic cancer cells. Exposure of cells with aqueous extract of Triphala for 24 h resulted in the significant reduced survival of cells in a dose-dependent manner with an IC_50 _of about 50 μg/ml (Fig 1A). In order to determine the mechanism of the antiproliferative effects of Triphala, experiments were carried out to measure the levels of cytoplasmic histone associated DNA fragments using cell death detection ELISA kit. Treatment of cells with 40 μg/ml or 60 μg/ml Triphala for 24 h resulted in increased number of apoptotic cells ranging from 2.9 to 6.0 folds over control (Fig 1B). To confirm the induction of apoptosis by Triphala, we determined the activation of caspase-3 and PARP in control and Triphala treated cells by western blotting. Treatment of Capan-2 cells with Triphala for 24 h caused significant activation of caspase-9, caspase-3 and PARP, as is apparent by the appearance of their cleaved products at 37 and 39 kDa (caspase-9), 19 and 17 kDa (caspase-3) and 89 kDa (PARP) (Fig 1C), suggesting that apoptosis induced by Triphala in these cells is mediated by caspase-3 cascade.

### Triphala causes DNA damage resulting in the activation of p53 in Capan-2 cells

**Figure 2:**
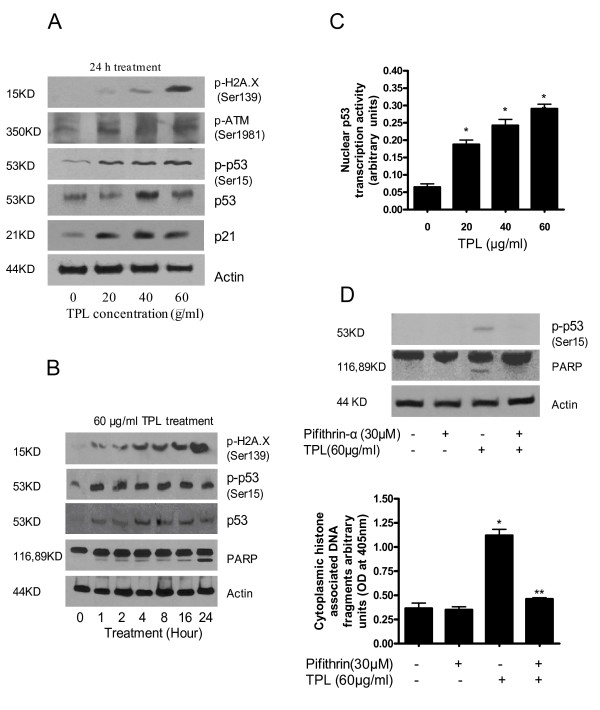
**Triphala causes DNA damage and activates p53 in Capan-2 cells.** A) Cells were treated with Triphala for 24 h and 60 μg total protein was separated by SDS-PAGE. The expression of p-H2A.X (Ser-139), p-ATM (Ser-1981), p-p53 (Ser-15), p53, and p21 were detected using appropriate antibodies. The blot was stripped and reprobed with the anti-actin antibody to ensure the equal protein loading. B) Time-dependent treatment cells with 60 μg/ml Triphala. C) Transcriptional activity of p53 in cells treated with Triphala for 24 h using TransAM p53 transcription assay kit. D) Effect of p53 inhibitor on Triphala induced apoptosis. Cells were pretreated with 30 μM pifithrin-α, p53 specific inhibitor for 1 hour followed by exposure to 60 μg/ml Triphala for 4 h in the presence of inhibitor. Apoptosis was determined by western blot or cell death detection kit. These assays were performed 2–3 times independently and obtained similar results. Statistical significance was determined by one-way ANOVA followed by Bonferroni's post hoc analysis for multiple comparisons. * Significantly different compared with PBS-treated control (P < 0.05). **Significantly different compared with Triphala treatment (P < 0.05).

Next we set out to investigate the mechanism of Triphala-induced apoptosis. We observed that Triphala treatment for 24 h caused significant phosphorylation of H2A.X at Ser-139 in a dose and time-dependent manner, suggesting the presence of DNA double strand breaks (Fig 2A–B). It is well known that in response to DNA damage, p53 is normally activated by ATM [22]. We observed that treatment of cells with Triphala caused subtle but statistically significant activation of ATM as evident by phosphorylation at Ser 1981 (Fig 2A). Our results further demonstrate that Triphala treatment resulted in the significant stabilization of p53 as evident by its phosphorylation at Ser-15 and increased protein level, in a dose and time dependent manner (Fig 2A–B). In fact, significant activation of p53 was noticed just after 1 h treatment with Triphala, which corroborated well with the DNA damage, also occurring at the same time (Fig 2B). Activation of p53 was further confirmed by evaluating the transcriptional activity of p53 in control and treated cells. As shown in Fig 2C, Triphala treatment for 24 h resulted in about 3 to 6 fold increased nuclear transcriptional activity of p53 in the cells as compared to control. Next we examined the expression of p21, which is the downstream molecule regulated by p53. Our results clearly indicate that Triphala treatment cause massive induction of p21 as compared to control (Fig 2A). To further confirm the involvement of p53 in Triphala-induced apoptosis, cells were pretreated with pifithrin-α (p53 specific inhibitor) prior to treatment with 60 μg/ml Triphala for 4 h. Pharmacologically blocking p53 activation almost completely abrogated Triphala-induced apoptosis as observed by PARP cleavage (Fig 2D) and ELISA based apoptosis assay (Fig 2D). These results indicate that apoptosis by Triphala in Capan-2 cells is mediated by p53 signaling pathway.

### Activation of ERK by Triphala

**Figure 3:**
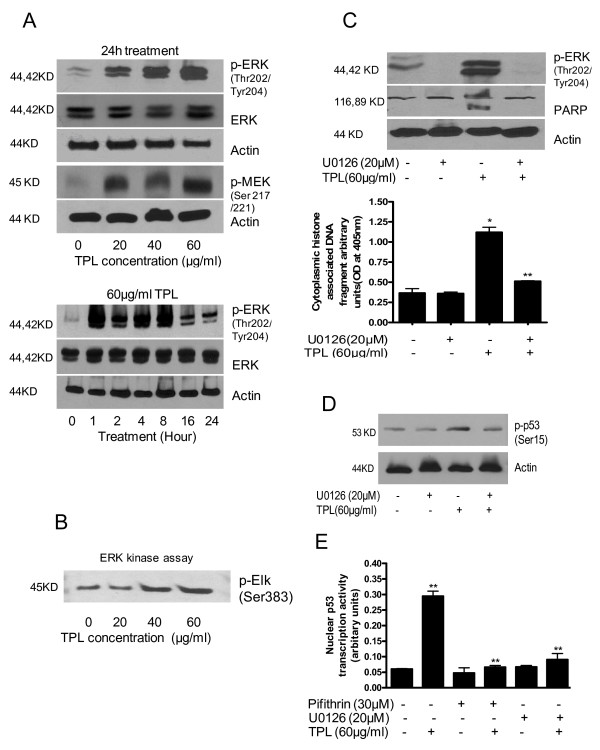
**Activation of ERK by Triphala**. A) Cells were treated with varying concentrations of Triphala for 24 h or with 60 μg/ml Triphala for different time intervals in a time-dependent experiment. Expression and phosphorylation of ERK (Thr-202/Tyr-204) and MEK-1 (Ser-217/221) was determined by western blotting. B) The effect of Triphala on the kinase activity of ERK was determined using a kit from Cell Signaling Technology, measuring the phosphorylation of Elk-1 at Ser-383. C, D) Effect of ERK inhibitor on Triphala induced apoptosis and activation of p53. Cells were pretreated with 20 μM U0126, MEK-1 inhibitor for 1 hour followed by exposure to 60 μg/ml Triphala for 4 h in the presence of inhibitor. Apoptosis and p53 was determined by western blot or using cell death detection kit. E) Effect of pifithrin and U0126 on the transcriptional activation of p53. These assays were performed 2–3 times independently with similar results. Statistical significance was determined by one-way ANOVA followed by Bonferroni's post hoc analysis for multiple comparisons. *Significantly different compared with PBS-treated control (P < 0.05). **Significantly different compared with Triphala treatment (P < 0.05).

Since we observed DNA damage and activation and stabilization of p53 by Triphala treatment, we next determined whether MAPK plays any role in p53 activation, as has been suggested in previous studies [23,24]. As shown in Fig 3A, treatment of cells with varying concentration of Triphala for 24 h caused considerable activation (phosphorylation) of ERK without causing any change at the protein level. In a time-dependent experiment, activation of ERK by 60 μg/ml Triphala was as early as 1 h and sustained for the duration of the experiment (Fig 3A). Triphala mediated activation of ERK was further verified by kinase activity of ERK by determining the phosphorylation of its downstream substrate Elk-1. Triphala caused increased phosphorylation of Elk-1 at Ser-383 in a dose-dependent manner (Fig 3B). In addition, Triphala caused phosphorylation of MEK-1 at Ser 217/221, which is the upstream regulator of ERK (Fig 3A). To further confirm the role of ERK in Triphala-induced apoptosis, cells were pretreated with MEK-1/2 inhibitor U0126 prior to treatment with 60 μg/ml Triphala for 4 h. As shown in Fig 3C, blocking ERK activation by U0126, nearly completely protected the cells from Triphala-induced apoptosis. These results clearly suggest that Triphala-induced apoptosis is mediated by ERK.

### DNA damage-induced activated ERK activates p53

ERK has been shown to get activated in response to DNA damage and further phosphorylate p53 [24]; however, this correlation is still not clearly established. In our experiments, we observed that both p53 and ERK get activated as early as 1 h after Triphala treatment. We therefore next wanted to determine whether ERK activates p53 in our system. Cells were pretreated with 20 μM MEK-1/2 inhibitor U0126 prior to treatment with Triphala for 4 h and then p53 was evaluated by western blotting and p53 transcriptional activity. Our results demonstrate that blocking ERK by U0126, partially blocked phosphorylation of p53 at Ser-15 (Fig 3D). However, U0126 completely blocked Triphala-induced p53 transcriptional activity as shown in Fig 3E. These results suggest that ERK may be upstream regulator of p53 in our model. Nevertheless, other pathways may also be functional in Triphala mediated DNA damaged cells leading to apoptosis.

### Triphala-induced ROS generation triggers ERK activation and apoptosis in Capan-2 cells

**Figure 4:**
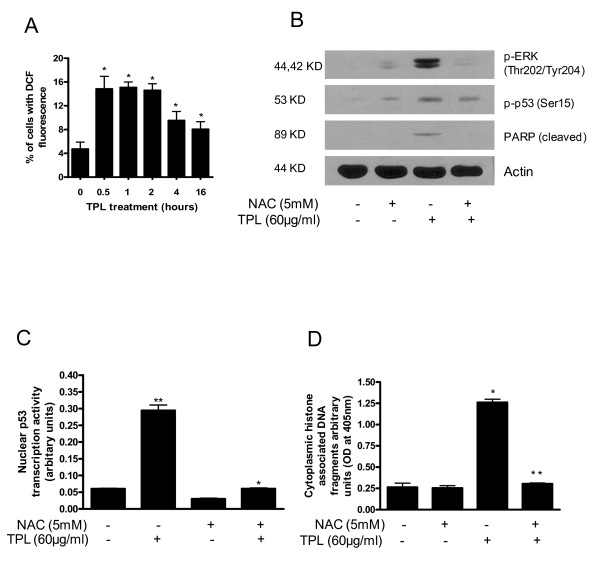
**Triphala causes ROS generation in Capan-2 cells.** A) Cells treated with 60 μg/ml Triphala for indicated time points were analyzed for DCF fluorescence (ROS generation) by flow cytometry after staining the cells with DCFDA. These experiments were repeated twice and obtained similar results. B) Activation of ERK, p53 and apoptosis by ROS can be abrogated by NAC. Cells were pretreated with 5 mM NAC for 1 hour and then treated with 60 μg/ml Triphala for 4 h. p-ERK, p-p53 and cleaved PARP were determined by western blotting as described in the method section. The blot was stripped and reprobed with anti-actin antibody to ensure equal protein loading. C) Effect of NAC on the transcriptional activity of p53. D) Apoptosis in NAC and Triphala treated cells was determined using cell death detection kit. Statistical significance in the treated group compared with control was analyzed by one-way ANOVA followed by Bonferroni's post hoc analysis. *Significantly different compared with PBS-treated control (P < 0.05). **Significantly different compared with Triphala treatment (P < 0.05).

Next important step was to determine the mechanism by which Triphala activates ERK and/or p53. Several studies including ours have implicated reactive oxygen species (ROS) as a possible mechanism for DNA damage and induction of apoptosis [20,21,25,26]. We therefore wanted to know whether Triphala-mediated activation of ERK, p53 and apoptosis in our model is associated with ROS generation. Generation of ROS was determined by flow cytometery in Capan-2 cells treated with 60 μg/ml Triphala at different time intervals. As shown in Fig 4A, Triphala treatment increased ROS generation over control as early as 0.5 h and sustained for the duration of the experiment. For example, 1 h treatment of cells with Triphala caused about 3.2 folds increase in ROS as compared to control. To investigate whether ROS generation contributes to activation of ERK and p53 and induction of apoptosis in our model, cells were pretreated with 5 mM antioxidant NAC prior to treatment with Triphala for 4 h. As shown in Fig 4B, NAC pretreatment almost completely blocked the activation of ERK induced by Triphala. P53 activation was however partially attenuated by NAC treatment (Fig 4B). Nonetheless, NAC pretreatment almost completely blocked Triphala-induced p53 transcriptional activity (Fig 4C). Furthermore, our results clearly demonstrate that NAC pretreatment conferred complete protection against Triphala-induced apoptosis as evaluated by PARP cleavage and histone-associated DNA fragmentation (Fig 4B, D). These results suggest that Triphala-mediated ROS may be responsible for ERK and/or p53 activation leading to induction of apoptosis.

### Effect of Triphala is not cell specific

**Figure 5:**
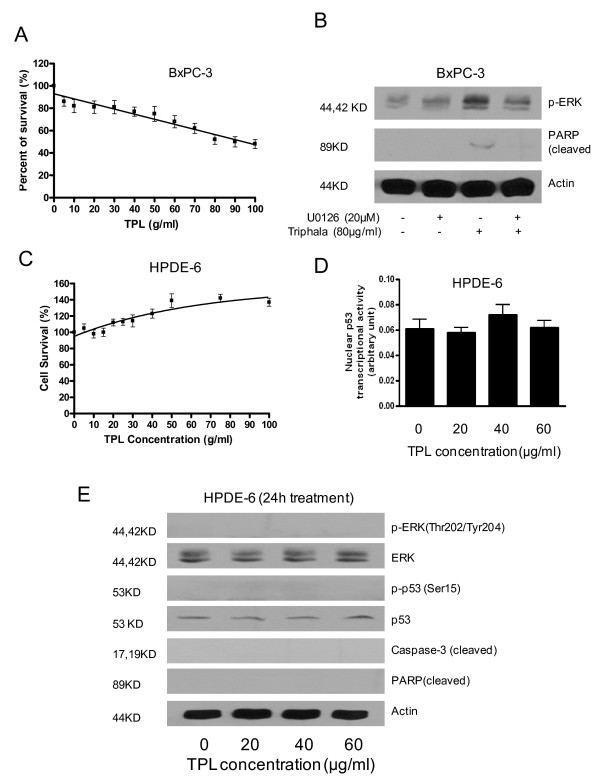
**Effect of Triphala is not cell specific.** A) Effect of Triphala was evaluated on the survival of BxPC-3 (mutated p53) pancreatic cancer cell line, as described in Method section. B) Effect of ERK inhibitor on Triphala induced apoptosis. Cells were pretreated with 20 μM U0126, for 1 hour followed by exposure to 80 μg/ml Triphala for 4 h in the presence of inhibitor. C) Effect of Triphala on the survival of HPDE-6, normal human pancreatic ductal epithelial cells. D) Transcriptional activity of p53 in HPDE-6 cells treated with Triphala for 24 h as described in Method section. E) Effect of Triphala on the activation of ERK, p53, caspase-3 and PARP in HPDE-6 cells. The blots was stripped and reprobed with anti-actin antibody to ensure equal protein loading. These assays were performed 2–3 times independently and obtained similar results. Statistical significance was determined by one-way ANOVA followed by Bonferroni's post hoc analysis for comparisons.

Effects of Triphala were also evaluated in BxPC-3 (harboring mutated p53) human pancreatic cancer cells and HPDE-6 (normal human pancreatic ductal epithelial) cells. Treatment of BxPC-3 cells with varying concentration of Triphala for 24 h resulted in the reduced survival of cells with an IC50 of about 85 μg/ml (Fig 5A). Similar to Capan-2 cells, Triphala treatment caused early and sustained activation of ERK in BxPC-3 cells (data not shown). Blocking ERK activation by U0126 completely blocked Triphala-induced apoptosis as shown by PARP cleavage (Fig 5B). On the other hand, Triphala failed to induce any cytotoxic effects on the survival of normal HPDE-6 cells (Fig 5C). Similarly, Triphala treatment did not caused any change in p53 transcriptional activity (Fig 5D) nor activated ERK or p53 and failed to activate caspase-3 and PARP in HPDE-6 cells (Fig 5E).

### Triphala inhibits the growth of Capan-2 human pancreatic tumor xenografts in vivo

**Figure 6:**
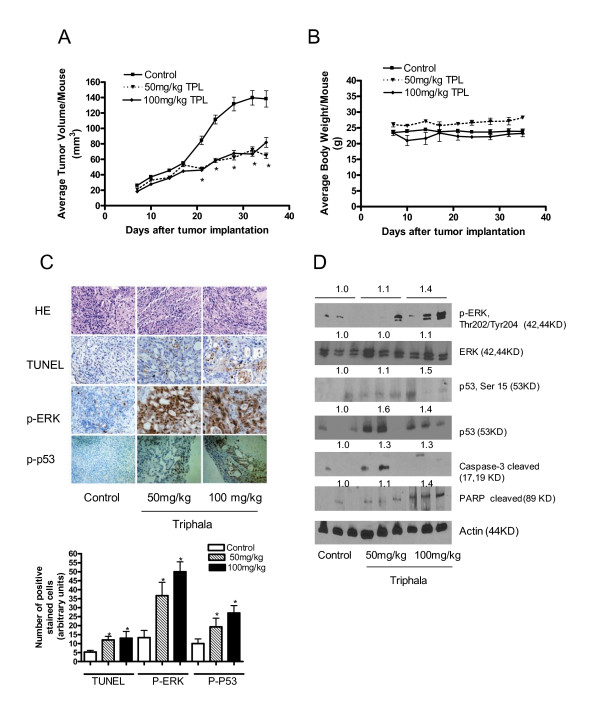
***In vivo *antitumor activity of Triphala in a xenograft model.** About 1 × 10^6 ^Capan-2 cells were injected subcutaneously on both left and right flanks of each athymic nude mouse and randomly assigned to three groups with 5 mice each group. Mice were fed with 50 mg/kg, or 100 mg/kg Triphala, 5 days/week by oral gavage. Control animals received PBS only. Tumors were measured by vernier calipers three times a week (Monday, Wednesday and Friday) once each mouse had palpable tumors. Each mouse was weighed twice a week (Monday and Friday). A) Average tumor volume in control (■), 50 mg/kg Triphala (▼) and 100 mg/kg Triphala treatment (◆). B) Average body weights of control and Triphala treated mice. Each data represents mean ± S.E. Error bars represent 95% confidence interval. *Statistically significantly different compared with control as analyzed by one-way ANOVA followed by Dunnett's test (P < 0.05). C) Tumor sections from control and Triphala treated mice were processed for H&E staining and immunohistochemistry for TUNEL, p-ERK and p-p53 staining. Each section was analyzed under the microscope at 200 × magnification and the number of stain positive cells were counted from three independent places on the slide. D) Immunoblot analysis for the expression of p-ERK (Thr-202/Tyr-204), p-p53 (Ser-15), ERK, and p53 using tumor lysates from control and Triphala treated mice. The blots were stripped and reprobed with actin antibody for equal protein loading. The numbers are the numerical representation of the mean densitometry values normalized to actin. These experiments were performed twice independently with similar results. Statistical significance was determined by one-way ANOVA followed by Bonferroni's post hoc analysis for comparisons.

The next most important step was to determine whether Triphala administration can suppress the growth of pancreatic tumor xenograft and whether Triphala causes apoptosis in the tumor cells *in vivo*. Based on our convincing *in vitro *results, we hypothesized that Triphala treatment would inhibit *in vivo *pancreatic tumor growth by activating ERK/p53 leading to apoptosis in the tumor cells. In order to test our hypothesis, pancreatic tumor xenografts were implanted in athymic nude mice by injecting 1 × 10^6 ^Capan-2 cells subcutaneously followed by administration of aqueous extract of 50 mg/kg or 100 mg/kg Triphala five days a week by oral gavage. The control animals received PBS only. Our results demonstrate that the growth of tumor was significantly inhibited in the mice that were treated with Triphala as compared with the growth of tumors in control mice (Fig 6A). For instance, at day 32 the average tumor volume in control mice was 139.7 ± 9.4 mm^3 ^as compared with 72.2 ± 4.0 mm^3 ^in 50 mg/kg or 66.9 ± 3.0 mm^3 ^in 100 mg/kg Triphala treated mice, which was approximately half the size of tumor in control mice (Fig 6A). The average body weight of control and Triphala treated mice did not changed significantly throughout the duration of the experiment (Fig 6B). Moreover, Triphala treated mice did not showed any signs of discomfort or impaired movement. These results suggest that both the doses of Triphala were equally effective in inhibiting the growth of Capan-2 xenograft.

### Triphala administration activated ERK, p53 and apoptosis in tumors

To further investigate the mechanism of reduced tumor growth by Triphala treatment, tumor tissues from control and Triphala treated mice were examined by immunohistochemistry and western blotting. Significantly higher counts of brown apoptotic bodies (as evaluated by TUNEL assay) were observed in the tumors from Triphala treated mice as compared with controls indicating that tumor growth inhibition in Triphala treated mice was due to increased apoptosis (Fig 6C). These results were further confirmed by western blot analysis of tumor lysates of control and Triphala treated mice. Cleaved fragments of caspase-3 and PARP were observed in the lysates of tumors from Triphala treated mice as compared to controls (Fig 6D). To gain further insight into the mechanism for increased apoptosis in response to Triphala treatment, we determined the activation of ERK and p53. As shown in Fig 6C, enormous staining of phospho-ERK was observed in the tumor sections from Triphala treated mice. Similarly, increased staining for phospho-p53 was also observed in response to Triphala treatment (Fig 6C). These results were further complemented by western blots where we observed increased phosphorylation of ERK without any change in the protein level in the tumors from Triphala treated mice. On the other hand, increased phosphorylation as well as protein expression of p53 was observed in Triphala treated tumors as compared to control tumors. These *in vivo *observations are in agreement with our *in vitro *data in Capan-2 cells. On the whole, our results indicate that Triphala mediated suppression of pancreatic tumor xenograft was associated with the activation of ERK and p53 leading to increased apoptosis in the tumor cells.

## Discussion

Triphala has been used for centuries in Ayurvedic medicine to treat various types of gastrointestinal-related disorders; however, the molecular mechanisms of Triphala have not been studied yet. In the present studies, we demonstrate that aqueous extract of Triphala is effective in inhibiting the growth of pancreatic cancer cells in culture as well as in the *in vivo *model. Our results reveal that Triphala treatment drastically reduces the survival of Capan-2 and BxPC-3 human pancreatic cancer cells in a dose-dependent manner. On the other hand, Triphala failed to cause any cytotoxic effects on the growth of HPDE-6 near normal pancreatic epithelial cells. Suppression of pancreatic cancer cell growth by Triphala in our model was due to induction of apoptosis, which in turn was associated with generation of ROS. Pretreatment of Capan-2 cells with antioxidant NAC blocked ROS generation and completely protected the cells from Triphala-induced apoptosis. Our results also demonstrate that Triphala treatment caused DNA damage resulting in the activation of ATM and ERK leading to stabilization of p53. Blocking ERK activation by MEK-1/2 inhibitor U0126 or p53 activation by pifithrin-α completely protected Capan-2 (wild type p53) cells from Triphala-induced apoptosis. Similarly, U0126 treatment blocked Triphala-induced apoptosis in BxPC-3 (mutated p53) cells, suggesting ERK as a molecular target of Triphala in pancreatic cancer cells. Further, orally feeding 50 mg/kg or 100 mg/kg Triphala to nude mice significantly retarded the growth of Capan-2 pancreatic tumor xenograft. Tumors from Triphala treated mice demonstrated increased apoptosis in the tumor cells, which was due to the activation of ERK and p53. To the best of our knowledge, this is the first study to report the molecular mechanism of the chemotherapeutic effects of Triphala against pancreatic cancer.

Reactive oxygen species (ROS) are the known mediators of intracellular signaling cascades. Excessive production of ROS nonetheless leads to oxidative stress, loss of cell function and apoptosis or necrosis [25-28]. Our results reveal that Triphala-induced apoptosis in pancreatic cancer cells is initiated by ROS generation, the effect of which can be blocked by antioxidant NAC. Several previous studies including those from our laboratory have implicated ROS as a possible mechanism for DNA damage and induction of apoptosis [26,28-31]. DNA damage plays a critical role in maintaining genomic integrity. Tumor cells exhibit genetic instability causing functional inactivation of p53 that plays an important role in DNA damage checkpoint pathways. In response to DNA damage, p53 is stabilized through phosphorylation at Ser 15 by ATM [22,32,33]. The effects of Triphala are compatible with this assertion. Our results do indicate that Triphala treatment causes DNA damage as depicted by increased phosphorylation of H2A.X at Ser 139, an indicator for the presence of DNA double-strand breaks.

DNA damage has been shown to activate the kinase activity of ATM, which subsequently modifies a number of downstream targets including phosphorylation of p53 at Ser 15 at the N-terminus [33,34]. Our studies reveal that Triphala treatment activates ATM by phosphorylation at Ser 1981. Moreover, our results also demonstrate increased protein expression and phosphorylation of p53 at Ser 15 in response to Triphala treatment. Stabilization of p53 by Triphala was further confirmed by nuclear transcriptional activity of p53. Induction of apoptosis by Triphala was almost completely blocked when the cells were pretreated with p53 specific inhibitor pifithrin, signifying the role of p53 in Triphala-induced apoptosis in pancreatic cancer cells.

A number of studies have shown the importance of ERK signaling pathway in regulating apoptosis [35-38]. Although, ERK pathway delivers a survival signal, quite a few recent studies have linked the activation of ERK with induction of apoptosis by various chemopreventive and chemotherapeutic agents [39-41]. In fact, oxidants have been shown to activate ERK by taking over the growth factor receptor signaling pathways [42-46]. Moreover, ERK may get activated in response to DNA damage and can phosphorylate p53 *in vitro *[23,24,47-49]. We found that exposure of Capan-2 or BxPC-3 cells with apoptosis-inducing concentration of Triphala results in a rapid and sustained activation of ERK in a concentration and time-dependent manner. Triphala mediated activation of ERK as well as apoptosis was completely abolished by MEK-1 inhibitor. MEK-1, which is an upstream of ERK, is also activated by Triphala in Capan-2 cells. Further, we observed that p53 is transcriptionaly regulated by ERK in response to Triphala treatment suggesting ERK as an upstream regulator of p53 in Capan-2 cells. We also observed that Triphala induce apoptosis by ERK activation in BxPC-3 cells, which has mutated p53. This is in part consistent with the observation that activated ERK lead to apoptosis after DNA damage in a p53 independent manner [49]. On the other hand, Triphala is not at all toxic to HPDE-6 normal pancreatic epithelial cells and does not activate ERK, p53 or caspases. Taken together, our results indicate ERK as a possible molecular target of Triphala in pancreatic cancer cells.

Pancreatic tumor growth inhibition and induction of apoptosis *in vivo *was observed by the oral administration of 50 mg/kg or 100 mg/kg Triphala 5 times a week. Our results are consistent with previous studies where Triphala was shown to be effective in suppressing the growth of Barc-95 (mouse thymic lymphoma) xenograft in mice [15]. Although, pharmacokinetics of Triphala in humans has not been determined, it has been used safely for centuries in the Ayuervedic medicinal system in India for the treatment of various gastrointestinal-related disorders. The effective dose of Triphala in our animal model for suppressing tumor growth, if extrapolated to humans ranges from 4 to 8 grams per day for a person weighing 70 kg. These doses of Triphala come within the dose range already being used by humans in countries such as India. The overall incidence of pancreatic cancer is approximately 8–10 cases per 100,000 persons per year in the USA [50-52]. In other countries the incidence may vary from 8–12 cases per 100,000 persons per year [51-53]. However, in some areas of the world, pancreatic cancer is sporadic; for instance, the incidence of pancreatic cancer in India is less than 2 cases per 100,000 persons per year [51-53]. It is tempting to speculate that the low incidence of pancreatic cancer in India may in part be due to consumption of Triphala or one of its constituent Amla (rich in ascorbic acid). Nevertheless, detailed epidemiological studies are required to substantiate this assumption. Interestingly, a very recent study demonstrated the anti-tumoral effects of ascorbic acid (component of Triphala) against ovarian, pancreatic and glioblastoma xenografts in mice [54]. Our recent studies have observed that Amla is also very effective against pancreatic cancer (manuscript communicated).

## Conclusion

Taken together, our studies demonstrate that Triphala-induced apoptosis in human pancreatic cancer cells is mediated by the activation of ERK and p53, which in turn is initiated by ROS generation. Moreover, oral administration of Triphala significantly suppress the growth of pancreatic tumor xenograft in nude mice by inducing apoptosis and activation of ERK and p53 in the tumor cells, a mechanism similar to that we observed *in vitro*. Our results thus provide a rationale for the development of Triphala as chemopreventive or chemotherapeutic agent against pancreatic cancer in the clinical practice.

## Competing interests

The authors declare that they have no competing interests.

## Authors' contributions

YS performed all major experiments and drafted the manuscript. RPS performed experiments during the revision of the manuscript. SKS was involved in the overall design of the study and writing the manuscript.

## Pre-publication history

The pre-publication history for this paper can be accessed here:


